# The Shehata technique for undescended testes that cannot be brought into the scrotum in one operation—a case series and meta-analysis

**DOI:** 10.3389/fped.2024.1371028

**Published:** 2024-04-19

**Authors:** Zhenying Lin, Zhongjing Yu, Huanyuan Li, Letu Wu ri ni, Baoxin Zhang

**Affiliations:** Department of Pediatric Surgery, Baoan Women’s and Children’s Hospital, Jinan University, Shenzhen, China

**Keywords:** Shehata technique, undescended testis, Fowler–Stephens orchiopexy, testicular atrophy, orchiopexy

## Abstract

**Background:**

Fowler–Stephens orchiopexy is commonly used for testes that cannot be brought into the scrotum in one operation. However, this surgical technique may result in a higher rate of testicular atrophy postoperatively.

**Methods:**

During the period between 2019 and 2023, we analyzed the cases of 20 patients in whom the Shehata technique was applied for testes that could not be brought into the scrotum in one operation, and we conducted a meta-analysis to explore the incidence of testicular atrophy vis-à-vis the Shehata technique and Fowler–Stephens orchiopexy.

**Results:**

The average age of the 20 patients was 3.78 (0.76–11.42) years. The blood supply to the testes was satisfactory, with the absence of atrophy, and the testes could be brought into the scrotum in stage II surgery. A postoperative reexamination with ultrasound revealed that the testes were securely positioned within the scrotum, with good blood supply and no atrophy, which was in contrast to their condition before the operation. The volume of the testes postoperatively was significantly greater than that of the preoperative testes (*p* = 0.009). There were no statistically significant differences in the growth rate of volume of the testes between the surgically treated side and the contralateral side (*p* = 0.25). The meta-analysis showed that the Shehata technique resulted in a lower incidence of testicular atrophy compared with Fowler–Stephens orchiopexy (*p* = 0.01).

**Conclusions:**

The Shehata technique preserves the main vessels of the testes with a lower incidence of testicular atrophy, which may be a valid and safe alternative to the Fowler–Stephens technique.

## 1 Introduction

Common surgical treatment techniques for an undescended testis include orchiopexy and Fowler–Stephens orchiopexy. Fowler–Stephens orchiopexy is commonly used for those testes that cannot be brought into the scrotum in one operation. However, this technique may induce a higher rate of testicular atrophy following surgery. In one study, postpubertal Wistar WU rats were used as models for Fowler–Stephens orchiopexy, which revealed significant decreases in the size of the testis, the diameter of the seminiferous tubules, and the normal sperm count. In addition, the pH levels in the tubules and interstitium also decreased, suggesting that testicular atrophy resulted from testicular ischemic injury (1). Therefore, there is still a clinical need to explore other potential surgical options for undescended testis. In another study, it has been reported that the Shehata technique can be used to treat high intra-abdominal testis without the need for vessel transection (2). This technique involves fixing the testis to a point 2.5 cm above and medial to the contralateral anterior superior iliac spine during stage I surgery (2). The authors reported that during stage II surgery, the sutures of the testis and the adhesions between the testis and the abdominal wall were removed, and then, the testis was also brought into the scrotum (2). The Shehata technique may be considered a better option for treating undescended testis that cannot be brought into the scrotum in one operation. In the context of the limited research on the Shehata technique for treating high cryptorchidism, in this study, we report a case series of 20 patients who were successfully treated with the Shehata technique for undescended testis that could not be brought into the scrotum in one operation. In addition, we conducted a meta-analysis to explore the incidence of testicular atrophy vis-à-vis the Shehata technique and Fowler–Stephens orchiopexy.

## 2 Materials and methods

### 2.1 Patients

This retrospective study was conducted at the Department of Pediatric Surgery, Baoan Women's and Children's Hospital, during the period between January 2019 and August 2023. The study was approved by the local Medical Ethics Committee and conformed to the principles of the Declaration of Helsinki. It is usually the case that if the classical laparoscopically assisted orchiopexy technique fails to bring the testis to the scrotum in a single operation, the Shehata technique is performed.

### 2.2 Inclusion criteria

The inclusion criteria were as follows: (1) patient age younger than 12 years; (2) an undescended testis that cannot be brought to the scrotum in one operation; (3) obtaining of informed consent from patients’ legal representatives.

### 2.3 Exclusion criteria

The exclusion criteria were as follows: (1) patient age of more than 12 years; (2) an undescended testis that can be brought to the scrotum in one operation; (3) failure to obtain informed consent; (4) incomplete data.

## 3 Surgical intervention

### 3.1 Stage I surgery

**Figure 1:**
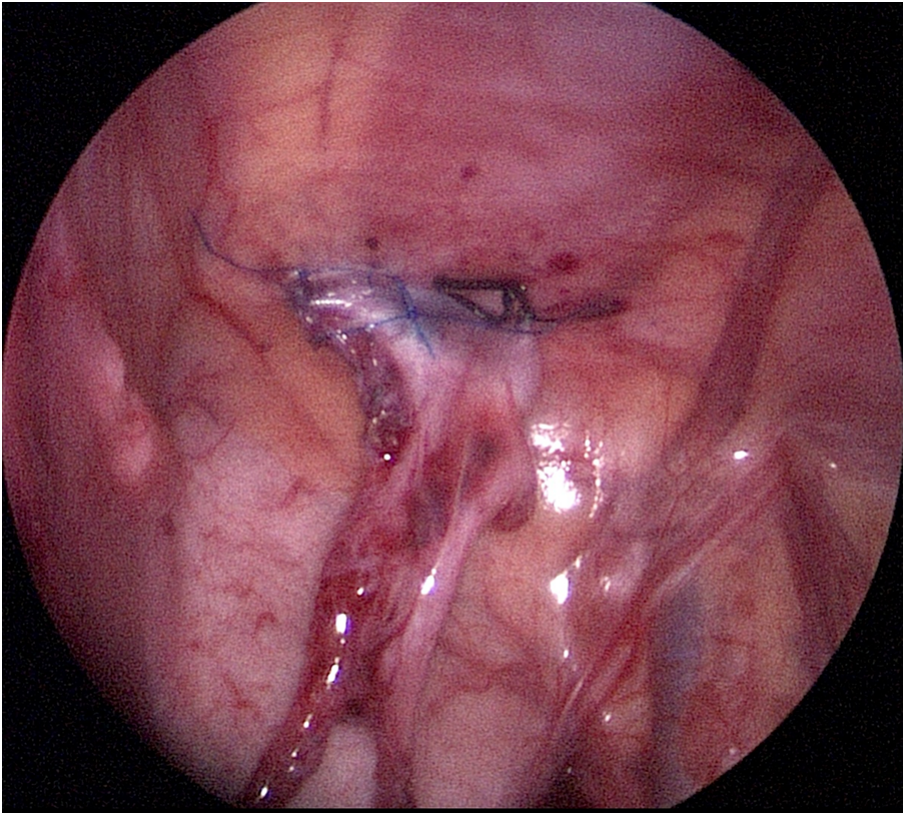
The affected testis was surgically fixed near the contralateral anterior superior iliac spine.

A skin incision was made at the superior border of the umbilicus, and initial peritoneal access was achieved with a Veress needle, followed by the insertion of a 3 mm trocar. Subsequently, two 3 mm trocars were inserted into the left and right midclavicular lines at the level of the umbilicus, respectively. The testis, vas deferens, and spermatic vessels were freed (freeing the spermatic vessels as high as possible). For patients who were unable to bring the testis to the contralateral internal ring without tension and who had an obviously insufficient visualized length of the spermatic cord, the Shehata technique was performed. Otherwise, we attempted an orchiopexy. A vascular clamp was passed from the scrotal incision into the abdomen. The testis was grasped at the gubernaculum tissue with the forceps and then brought out and placed into the subdartos pouch. If the testis could not reach the ipsilateral scrotum without tension, the Shehata technique was also performed. A non-absorbable suture was passed trans-abdominally into the abdominal cavity on a curved needle, avoiding the testicular vessels and vas deferens, and fixing the testis to a point above and medial to the contralateral anterior superior iliac spine twice. However, for a small number of patients with severely insufficient spermatic vessel lengths, the testis was fixed in the area between the bladder and the contralateral anterior superior iliac spine (Figure 1).

### 3.2 Stage II surgery

Removal of the sutures from the testis and the adhesions between the testis and the abdominal wall was performed. The affected testicular vessels and vas deferens were mobilized when their lengths were found to be insufficient. Then, the testis was brought into the scrotum without tension.

## 4 Literature review

A search of the literature (up to 30 July 2023) using PubMed, Cochrane Review database, Embase, China National Knowledge Infrastructure, and Wanfang was performed to identify studies that estimated the clinical effects of the Shehata technique in treating high cryptorchidism. Searches were performed using the terms “Shehata” or “Staged laparoscopic traction-orchiopexy.” We also searched the reference lists of the relevant literature. Data were initially extracted and evaluated independently by two investigators.

## 5 Statistical analysis

The clinical data were analyzed using the R software. The formula for estimating the volume (V) of the testis, based on the measurements of length (l) and width (w) using ultrasound, is V = l × w^2^ × 0.52 (3). All data on testicular volume were tested for normality using the Shapiro–Wilk test. If the data followed a normal distribution pattern, a paired sample *t*-test was performed. If the data were not normally distributed, a Wilcoxon paired rank-sum test was conducted. A meta-analysis was performed using Review Manager Software 5.4 (Review Manager, Nordic Cochrane Centre, UK) to compare the incidence of testicular atrophy between the Shehata technique and Fowler–Stephens orchiopexy. Pooled odds ratios (ORs) and 95% confidence intervals (CIs) were calculated for the dichotomous outcomes. The *I*^2^ statistic test and Chi-squared test were performed to assess heterogeneity. A value of *I*^2^ > 50% was regarded as indicating substantial heterogeneity. The fixed-effects model was used when *I*^2^ < 50%; otherwise, the random-effects model was used. A *p*-value < 0.05 was considered statistically significant.

## 6 Results

**Figure 2:**
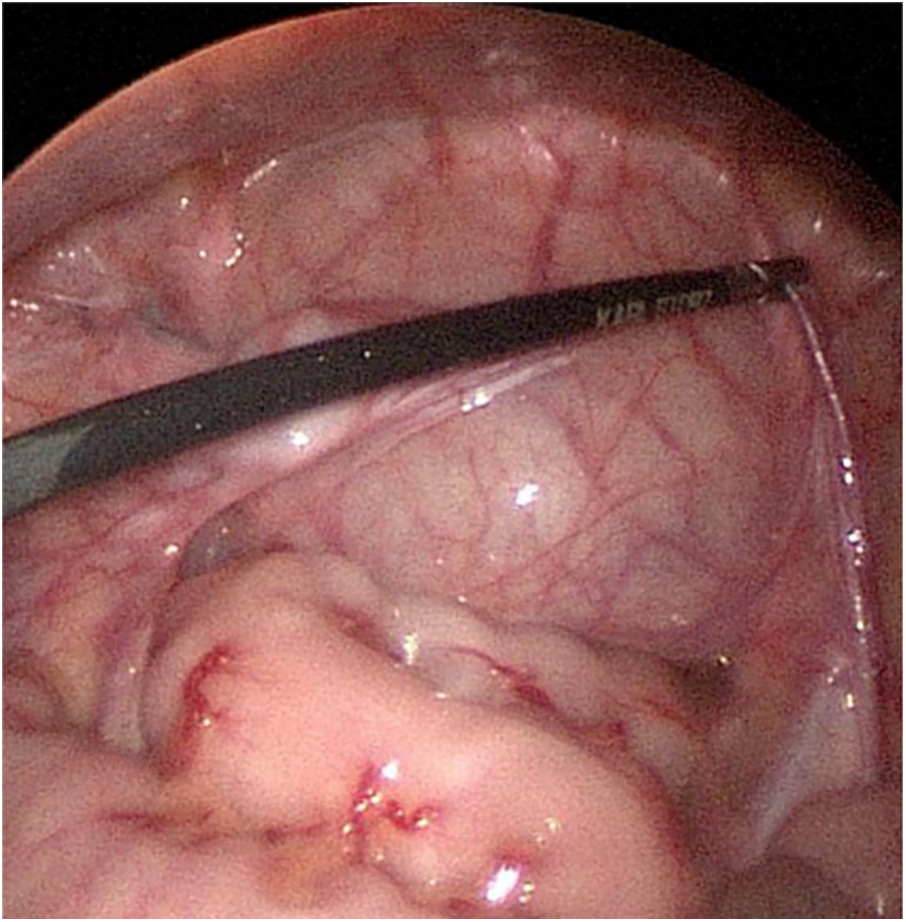
An elongation of the testicular vessels and the vas deferens was found during the secondary operation.

A total of 20 patients met our inclusion criteria and were included in the analysis. The average age of these patients was 3.78 years, which ranged from 0.76 to 11.42 years. The mean interval between the two stages of surgery was 6.01 months, which ranged from 3.91 to 12.65 months. In all the 20 patients, the testes did not reach the ipsilateral scrotum without tension during stage I surgery. During stage II surgery, the lengths of their testicular vessels and vas deferens were found to be longer than before surgery (Figure 2). In addition, we observed that their testes were normal and could reach the ipsilateral scrotum without tension.

After stage I surgery and before stage II surgery, all patients were followed up for a mean period of 5.71 months (ranging from 3.19 to 12.9 months). During this time, an evaluation through color Doppler ultrasound was conducted, and the test results indicated that blood supply to the testes was good. No complications such as adhesive intestinal obstruction or internal hernia behind the testicular vessels were observed. Following stage II surgery, all patients underwent a follow-up for a mean period of 6.13 months (ranging from 3 days to 30 months). This follow-up included an evaluation of symptoms and ultrasound scan results, which confirmed that the testes were fixed in the scrotum. No testicular atrophy or other complications were observed, and blood supply to the testes remained good. The mean follow-up period for all 20 patients was 26.05 months (ranging from 2.53 to 48.59 months), and the follow-up was conducted via telephone or outpatient visits. We found no evidence of any abnormalities, aberrant locations of testes, or discomfort or pain in the testes.

The Shapiro–Wilk test indicated that the data within the study groups were not normally distributed; therefore, the Wilcoxon matched-pairs signed-rank test was performed. This test revealed that the volume of the postoperative testes was significantly greater than that of the preoperative testes (*p *= 0.009). There were no statistically significant differences in the growth rate of the testes’ volume between the surgical side and the contralateral side (*p* = 0.25).

### 6.1 Meta-analysis

**Figure 3:**
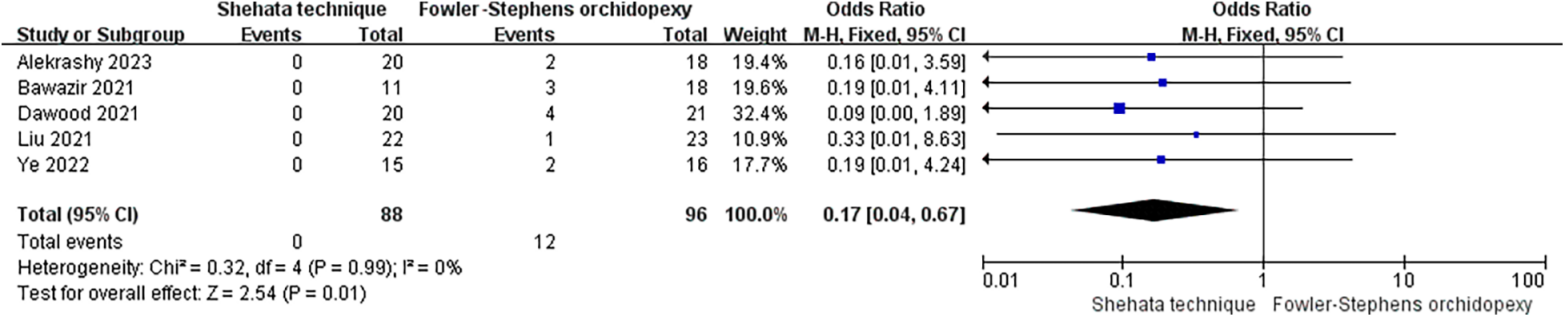
A comparison of the incidence of testicular atrophy between the Shehata technique group of patients and the Fowler–Stephens orchiopexy group of patients.

A total of five studies involving 88 patients treated with the Shehata technique and 96 treated with Fowler–Stephens orchiopexy met the inclusion criteria (4–8). The meta-analysis showed that the Shehata technique resulted in a lower incidence of testicular atrophy compared with Fowler–Stephens orchiopexy (OR = 0.17, 95% CI 0.04, 0.67; *p* = 0.01), and there was no significant statistical heterogeneity (*I*^2^ = 0.0%, *p* = 0.99) (Figure 3).

## 7 Discussion

Undescended testis is a common congenital disorder in which unilateral or bilateral testes fail to descend into the scrotum. It may lead to complications such as testicular atrophy, infertility, testicular torsion, and malignant transformation (9). Therefore, active treatment for undescended testis is necessary. The intra-abdominal testis can be located anywhere from the lower pole of the kidney to the lower end of the internal inguinal canal. Surgical treatments for undescended testis include orchiopexy and Fowler–Stephens orchiopexy. Docimo reviewed and analyzed 8,425 cases of patients with undescended testis from 64 articles and found that the success rate for Fowler–Stephens orchiopexy was 67%, and for staged Fowler–Stephens orchiopexy, it was 77% (10). Esposito et al. performed the two-step Fowler–Stephens procedure using the laparoscopy technique on 12 patients. Among these, 16.7% experienced atrophy of the testis in the scrotum, whereas 83.3% retained a viable testis in the scrotum. Despite the good vascularization observed on echo color Doppler ultrasound, the operated testis was consistently smaller than the normal, unoperated one (11). Hvistendahl and Poulsen reported that a total of 65 patients with intra-abdominal testes underwent the laparoscopic two-stage Fowler–Stephens procedure. Of these patients, 60 required two-stage operations, while in five, the testes were removed during the second stage because of testicular atrophy or a short vas deferens. The procedure was successful in 80% of the patients (12). Yu et al. reviewed 60 studies, which included a total of 1991 operated testes. The overall success rates for one-stage and two-stage Fowler–Stephens orchiopexies were 85% and 87%, respectively. In addition, the overall atrophy rate was 10% (13). The success rates for one-stage Fowler–Stephens orchiopexies, both open and laparoscopic, were 83% and 87%, respectively, with atrophy rates of 12% and 8%. The corresponding success rates for two-stage Fowler–Stephens orchiopexies were 81% and 89%, respectively, with atrophy rates of 17% and 8% (13). When evaluating laparoscopic Fowler–Stephens orchiopexy over time, the success rates prior to the year 2000, between 2000 and 2010, and after 2010 were 85%, 89%, and 88%, respectively, with atrophy rates of 15%, 9%, and 6% (13). In 2016, it was reported that the success rate for the Shehata technique was 84% (2). The viability of the testicles relies on the presence of collateral blood supply from the vessels in the inguinal canal, hypertrophy of the artery of the vas deferens, and gubernacular vessels (14). Considering that Fowler–Stephens orchiopexy requires vessel ligation and may induce a higher rate of testicular atrophy after surgery, the Shehata technique dissociates the spermatic cord blood vessels without cutting them. This theoretically protects the blood vessels in the testicles and uses the bowel to slowly pull and lengthen the testicular vessels and vas deferens, allowing the blood vessels in the spermatic cord to extend gradually. This helps prevent testicular ischemia that may be caused by forced pulling. Therefore, the Shehata technique was performed in this study, and no testicular atrophy was found in the regular reexamination under color Doppler ultrasound. The testicular vessels and vas deferens were found to be longer than before surgery. A postoperative reexamination with color Doppler ultrasound showed good testicular blood supply without testicular atrophy. A statistical analysis showed that the volume of the postoperative testes was significantly higher than that of the preoperative testes, and there were no statistically significant differences in the growth rate of the volume of the testes between the surgical side and the contralateral side. A meta-analysis showed that the Shehata technique resulted in a lower incidence of testicular atrophy compared with Fowler–Stephens orchiopexy.

Shehata et al. included cases of patients with intra-abdominal testis occurring between the ages of 6 months and 9 years (2). However, in this study, we expanded the age range of our patients to include those between 9 and 12 years old.

Shehata et al. excluded cases of patients in whom the testis had reached the contralateral internal inguinal ring without tension (2). We adjusted the inclusion criteria for the Shehata technique to apply it only to those patients who were unable to bring the testis to the contralateral internal ring without tension and who exhibited a clearly insufficient visualized length of the spermatic cord. In such patients, the Shehata technique was employed. If the testis could not be brought down to the scrotum without tension, we performed the Shehata procedure. Conversely, if the testis could be positioned in the scrotum without tension, the Shehata procedure was deemed unnecessary, sparing the patient an additional surgery.

In this surgical technique, the majority of testicles were fixed near the contralateral anterior superior iliac spine, approximately 2.5 cm above it, with appropriate tension. However, for a small number of patients with severe deficiency of the spermatic vessels, fixing the testis near the contralateral anterior superior iliac spine was not possible. In such patients, the testis was fixed in the area between the bladder and the contralateral anterior superior iliac spine, which also yielded satisfactory results.

Shehata et al. reported 140 cases of surgery, with 16 cases of testis dropped from the fixation position (2). Dawood et al. reported 20 testes cases of surgery, with 2 cases of testis dropped from the fixation position (4). Bawazir et al. reported 11 cases of surgery, with 3 cases of testis dropped from the fixation position (7). Elsherbeny et al. reported 20 cases of surgery, with 2 cases of testis dropped from the fixation position (14). However, in our surgeries, no instances of the testis moving out of the fixation position were observed, which may be attributed to the simultaneous fixation of the testicle and the gubernaculum during stage I surgery. Another possible explanation could be the limited number of surgical cases.

This study has some limitations. Because of the difficulty patient guardians experience in fully complying with the doctor's advice, there is an obvious difference in the intervals between surgeries for different children. Furthermore, the duration of postoperative follow-up is not consistent across patients. In addition, the small proportion of children requiring staged surgery for cryptorchidism contributes to a limited clinical sample size.

## 8 Conclusion

The Shehata technique preserves the main vessels of the testes with a lower incidence of testicular atrophy, and may be a valid and safe alternative to the Fowler–Stephens technique.

## Data Availability

The raw data supporting the conclusions of this article will be made available by the authors without undue reservation.
